# The Influence of Different Extraction Techniques on the Chemical Profile and Biological Properties of *Oroxylum indicum*: Multifunctional Aspects for Potential Pharmaceutical Applications

**DOI:** 10.1155/2022/8975320

**Published:** 2022-09-19

**Authors:** Kai Yan, Xin-jie Cheng, Guang-li Bian, Yan-xia Gao, De-qiang Li

**Affiliations:** ^1^Hebei Institute for Drug and Medical Device Control, No. 219 Yuquan Road, Shijiazhuang 050227, Hebei Province, China; ^2^Department of Pharmacy, The Second Hospital of Hebei Medical University, No. 215, Heping West Road, Shijiazhuang 050000, Hebei Province, China

## Abstract

*Oroxylum indicum* (L.) Kurz (Bignoniaceae), a traditional Chinese herbal medicine, possesses various biological activities including antioxidant, anti-inflammatory, antibacterial, and anticancer. In order to guide the practical application of *O. indicum* in the pharmaceutical, food, and cosmetic industries, we evaluated the effects of five different extraction techniques (maceration extraction (ME), oxhlet extraction (SOXE), ultrasound-assisted extraction (UAE), tissue-smashing extraction (TSE), and accelerated-solvent extraction (ASE)) with 70% ethanol as the solvent on the phytochemical properties and biological potential. The UHPLC-DAD Orbitrap Elite MS technique was applied to characterize the main flavonoids in the extracts. Simultaneously, the antioxidant and enzyme inhibitory activities of the tested extracts were analyzed. SOXE extract showed the highest total phenolic content (TPC, 50.99 ± 1.78 mg GAE/g extract), while ASE extract displayed the highest total flavonoid content (TFC, 34.92 ± 0.38 mg RE/g extract), which displayed significant correlation with antioxidant activity. The extract obtained using UAE was the most potent inhibitor of tyrosinase (IC_50_: 16.57 ± 0.53 mg·mL^−1^), while SOXE extract showed the highest activity against *α*-glucosidase (IC_50_: 1.23 ± 0.09 mg·mL^−1^), succeeded by UAE, ME, ASE, and TSE extract. In addition, multivariate analysis suggested that different extraction techniques could significantly affect the phytochemical properties and biological activities of *O. indicum*. To sum up, *O. indicum* displayed expected biological potential and the data collected in this study could provide an experimental basis for further investigation in practical applications.

## 1. Introduction

Natural products with unique pharmacological activities, especially traditional Chinese medicine (TCM), are considered to be a treasure trove of resources for new drugs, functional foods, and dietary supplements [1, 2]. TCM is rich in bioactive compounds, such as flavonoids, polyphenols, and alkaloids. These compounds may contribute to a wide variety of pharmacological activities [3]. Searching for bioactive substances from natural products as nutrients and functional food ingredients has been recently accepted by a growing number of people and has gradually become a research hotspot in related fields due to people's attention to health and medication safety, as well as the prominent advantages of natural products in the treatment and prevention of diseases [4].

*Oroxylum indicum* is the dried mature seed of *Oroxylum indicum* (L.) Kurz and belongs to the family Bignoniaceae. It is widely distributed in China, India, and other Asian countries [5]. It has been used in folk remedies for centuries with a certain medicinal value, which is mainly used to treat cough-based respiratory diseases [6]. Modern pharmacological studies have demonstrated that *O. indicum* has a wide spectrum of biological activities, including antioxidant [7, 8], anti-inflammatory [9], antibacterial [10], analgesic [11], anticancer [12], and antidiabetic properties [13, 14]. Phytochemical research indicates that flavonoids are the main chemical components of *O. indicum* [13]. Recent studies have shown that flavonoids contribute to biological activities, so they are the main substances for *O. indicum* to exert biological activities [13]. Owing to its potential nutritional value, this plant is widely used in the food, drug, health care products, cosmetics, and functional beverage industries [6].

The extraction process is a key step in the preparation of natural products and the analysis of bioactive compounds [15, 16]. It is well-established that extraction of specific bioactive compounds from natural products remains a challenge due to the complex chemical components, a large number of interfering substances, low content of active substances, and large differences in physicochemical properties [17]. Regarding extraction technologies, the extraction of bioactive substances from natural products is mainly based on conventional extraction techniques, such as maceration extraction (ME) and Soxhlet extraction (SOXE). Due to the relatively complete extraction, SOXE is widely used to isolate bioactive phytochemicals. Although this method has the advantage of high yield, it also has disadvantages including time-consuming, large volume of solvent, and low efficiency. ME has the defects of low leaching rate and is time-consuming as a traditional extraction method, but the component damage is difficult to achieve. Numerous nonconventional extraction techniques with shorter extraction times, higher extraction efficiency, and higher active ingredient content have emerged with the development of science and technology [18, 19]. The superiority of ultrasound-assisted extraction (UAE) includes high extraction efficiency, short time, low temperature, wide adaptability, and simplicity [18]. In addition to the benefits mentioned above, tissue-smashing extraction (TSE) can fully protect the heat-sensitive components, and the whole process is green and environmentally friendly [20]. Accelerated-solvent extraction (ASE) has the advantages of less organic solvent consumption, rapidity, less matrix influence, high recovery rate, and good reproducibility [19]. However, exposure to high temperature and pressure may result in destruction of certain components. When it comes to time consumption, cost, extraction efficiency, and environmental impact, each extraction method shows its own merits and drawbacks [21]. In addition, there are differences in the composition and content of bioactive substances in extracts obtained by different extraction processes, resulting in certain differences in biological activities [22–24]. In other words, the characteristics of extracts depend on the choice of extraction procedures [25]. Thus, it is necessary to select an appropriate extraction method to obtain high content of bioactive substances from naturally-derived plant materials and fully explore their potential applications.

Therefore, this study is aimed to provide a comprehensive analysis of the phytochemical components and potential biological activities of *O. indicum* extracts obtained by different extraction techniques. The chemical constituents of *O. indicum* were identified by UHPLC-DAD Orbitrap Elite MS, and four main flavonoids were quantitatively analyzed by UHPLC. The total bioactive compounds, antioxidant activities, and enzyme inhibitory activities were determined simultaneously. Besides, the differences in chemical profiles and biological properties brought about by extraction methods were performed by multivariate analysis. This study clarified the application prospects of *O. indicum* in the functional food, drug, nutraceutical, and cosmetics industries.

## 2. Materials and Methods

### 2.1. Chemicals and Reagents

The standard of rutin was supplied by the National Institutes for Food and Drug Control (Beijing, China). Oroxin A, oroxin B, baicalein, chrysin, and oroxylin A were purchased from Must Biotechnology Co. Ltd (Chengdu, China). Gallic acid was purchased from Solarbio Science & Technology Co. Ltd (Beijing, China). *α*-glucosidase (EC 3.2.1.20, 32.4 U·mg^−1^), mushroom tyrosinase (EC 1.14.18.1, 1560 U·mg^−1^), L-DOPA, and 4-nitrophenyl-*β*-D-glucopyranoside (PNPG) were purchased from Baoman Biotechnology Co. Ltd (Shanghai, China). 2,2′-azinobis (3-ethylbenzothiazoline-6-sulfonic acid ammonium salt (ABTS), (±)-6-hydroxy-2,5,7,8-tetramethylchromane-2-carboxylic acid (Trolox), and neocuproine was purchased from Aladdin Biochemical Technology Co. Ltd (Shanghai, China). 2,2-diphenyl-1-picrylhydrazyl (DPPH) was purchased from ApexBio Technology LLC (Houston, USA). 2,4,6-Tri(2-pyridyl)-1,3,5-triazine (TPTZ) and Folin & Ciocalteu's phenol reagent were purchased from Solarbio Science & Technology Co. Ltd (Beijing, China). Formic acid (analytical grade) was purchased from Thermo Fisher Technology Co. Ltd (Shanghai, China). HPLC-grad methanol, ethanol, and acetonitrile were obtained from Merck (Darmstadt, Germany). The ultrapure water used in this study was obtained by a Milli-Q water purification system (Millipore, Billerica, MA, USA). All other chemicals and reagents used were of analytical grade.

### 2.2. Plant Materials

*Oroxylum indicum* was purchased from Nanning, Guilin Province, China. The species was identified as *Oroxylum indicum* (L.) Vent by Dr. Yong-li Liu (Hebei Institute for Drug and Medical Device Control, Shijiazhuang, Hebei, China). The voucher specimens were deposited at the Department of Pharmacy, the Second Hospital of Hebei Medical University, Shijiazhuang, Hebei, China.

### 2.3. Extracts Preparations

#### 2.3.1. Maceration Extraction (ME)

To obtain maceration extract, one gram of crushed seeds was macerated with 30 mL of ethanol : water (70 : 30, v/v) at room temperature in the dark for 24 h.

#### 2.3.2. Soxhlet Extraction (SOXE)

The powdered seeds (2 g) were placed on the filter paper and extracted with 70% ethanol solution (60 mL, 1 : 30 ratio) in a Soxhlet apparatus for 4 h at 100°C.

#### 2.3.3. Ultrasound-Assisted Extraction (UAE)

1 g powdered *O. indicum* was mixed with 30 mL of ethanol-water solution (70%, v/v). The mixture was sonicated at 30°C by a SB-5200DT ultrasonic device (Ningbo, Zhejiang, China) operating at a power of 300 W and a frequency of 40 kHz for 30 min.

#### 2.3.4. Tissue-Smashing Extraction (TSE)

0.2 g of crushed seeds were extracted by a dispersing machine using 70% aqueous ethanol (1 : 30 ratio of plant material to aqueous ethanol, w/v) as an extraction solvent at 25600 rpm for 1 min to prepare TSE samples.

#### 2.3.5. Accelerated-Solvent Extraction (ASE)

ASE was carried out using BUCHI SpeedExtractor E-916 instrument (Flawil, Switzerland). 1 g powdered seeds was mixed with diatomic Earth thoroughly in 40 mL extraction cell and extracted with 70% aqueous ethanol. The extractions were performed at 100°C with a pressure of 100 bar, then heated for 1 min and maintained for 5 min, continuing for two cycles. The extraction solvent (2 min) and N_2_ (5 min) were used to flush the extraction cell, and extracts obtained were collected into the collection flask finally.

All obtained extracts were centrifuged at 10,000 × *g* for 10 min at room temperature before the supernatant was filtered with a 0.22 *μ*m microporous membrane. All samples were stored at 4°C for subsequent analysis.

### 2.4. UHPLC-DAD Orbitrap Elite MS Analysis of the Extracts

Analysis of the extracts was carried out by an UHPLC system hyphenated to an Orbitrap Elite mass spectrometer (Thermo Fisher SCIENTIFIC, Bremen, Germany) on which a chromatographic separation on a Kinetex-C_18_ (4.6 × 100 mm, 2.6 *μ*m) column was performed. The mobile phase was composed of 0.1% formic acid aqueous solution (A) and acetonitrile (B) with a flow rate of 0.3 mL/min at 30°C. The optimized gradient elution program was as follows: 0–15 min, 25%–35% B; 15–20 min, 35%–70% B; 20–30 min, and 70% B; then, the initial mobile phase, 25% B, was recovered within 1 min and maintained for 5 min to equilibrate the column. A diode array detector (DAD) at 277 nm was applied to monitor the effluents from UPLC, and the injection volume was set at 5 *μ*L.

Mass spectrometric detection was conducted on an Orbitrap Elite system with a heated electrospray ionization (HESI) source in the negative ion mode. The ion spray voltage was 3.6 kV. The capillary and atomizer temperatures were both set at 350°C; and the sheath gas and auxiliary gas were 4.5 L/mL and 6.5 L/mL, respectively. Nitrogen and high-purity helium were used as atomizing gasses and collision gasses, respectively. The scanning mode was set as full scan/data-dependent two-level scan (Full MS/dd-MS2) mode, in which the resolution of the first-level Full MS full scan was 60000, while the secondary scanning resolution of dd-MS2 was 15000. The scan range was from *m*/*z* 50 to 1000. Additionally, Xcalibur software (version 2.1, Thermo Fisher Scientific, Waltham, MA, USA) was used for instrument control, data acquisition, and data analysis.

### 2.5. Determination of Bioactive Compounds

#### 2.5.1. Total Phenolics Content (TPC)

In this study, TPC was determined by the Folin–Ciocalteu method given in a previous study with some modifications [26]. Briefly, 0.1 mL of the tested sample was placed in a 10 mL volumetric flask and thoroughly mixed with 0.5 mL Folin–Ciocalteu reagent. After 3 min, 2 mL of 15% Na_2_CO_3_ was added to the mixture, and then diluted to the mark with ultrapure water. The blend was incubated at room temperature in the dark for another 1 h. The absorbance of 0.2 mL of the reaction liquid was measured at 765 nm with a SpectraMax M2 Multifunctional microplate reader (Sunnyvale, CA, USA). Gallic acid was employed for comparison, and TPC was expressed as mg of gallic acid equivalents (mg GAE/g extract).

#### 2.5.2. Total Flavonoids Content (TFC)

NaNO_2_-Al(NO_3_)_3_ method was used for TFC determination with some modifications [27]. In brief, 0.5 mL of the extract was measured precisely and placed in a 10 mL volumetric flask. First, 0.5 mL of 5% NaNO_2_ and 10% Al(NO_3_)_3_ solution were added to the volumetric flask successively at an interval of 6 min, and the solution was kept for another 6 min. Then, 4% NaOH (5 mL) was added at a constant volume of 10 mL with water. After 15 min, 0.2 mL of the test samples were placed in a 96-well plate, and the absorbance was measured at 510 nm. All operations were performed at room temperature. Rutin was used as the reference compound for measuring TFC and the results were expressed as mg of rutin equivalents (mg RE/g extract).

### 2.6. Determination of Antioxidant Activities

We researched the antioxidant activities of extracts obtained from different extraction methods. A Trolox (a hydrophilic analogue of vitamin E) standard curve was used as a calibration standard to determine the antioxidant activities of extracts. The extracts were diluted ten times for antioxidant activity analysis.

#### 2.6.1. Assay of ABTS Scavenging Activity

ABTS scavenging activity was determined according to a previously reported method with slight modifications [28]. The same volume of 7 mM ABTS and 2.45 mM potassium persulfate was mixed to obtain the free radical solution. The blend was incubated for 12–16 h in the dark at room temperature before use. The mixture was then diluted with ethanol until the absorbance was 0.70 ± 0.02 at 734 nm. 180 *μ*L of diluted radical solution was added to the microplate well, which was followed by the addition of 20 *μ*L extraction solution. After the mixture was kept in the dark for 6 min at room temperature, the absorbance was read at 734 nm. The capability of ABTS scavenging activity was calculated by the following equation:

(1) $$\begin{array}{c} \text{ABTS scavenging ability}\left(\%\right)=\left[\frac{A_{0}-\left(A_{1}-A_{2}\right)}{A_{0}}\right]\times 100\%, \end{array}$$

where *A*_0_ is the absorbance value of the blank without extract, *A*_1_ is the absorbance value of extract, and *A*_2_ is the absorbance value of the control without ABTS.

#### 2.6.2. Assay of DPPH Radical Scavenging Activity

The DPPH radical scavenging activity was analyzed based on a method described by Wu et al. with minor modifications [29]. Then, 180 *μ*L of 0.2 mM DPPH solution and 20 *μ*L extraction solution were added to a 96-well plate, and the reaction solution was protected from light for 30 min at room temperature, after which the absorbance value was determined at a wavelength of 517 nm. The calculation formula for the scavenging capability of DPPH was as follows:

(2) $$\begin{array}{c} \text{DPPH scavenging ability}\left(\%\right)=\left[\frac{A_{0}-\left(A_{1}-A_{2}\right)}{A_{0}}\right]\times 100\%, \end{array}$$

where *A*_0_, *A*_1_, and *A*_2_ are the absorbance value of the blank without extract, extract, and the control without DPPH, respectively.

#### 2.6.3. Ferric Reducing Antioxidant Power (FRAP)

The FRAP was assayed following the method described by Daniel et al. with some modifications [30]. The FRAP reagent was composed of 2,4,6-Tri(2-pyridyl)-1,3,5-triazine (TPTZ) solution (10 mM in 40 mM HCl), FeCl_3_ (20 mM) and acetate buffer (0.3 mM, pH 3.6) mixed in a ratio of 1 : 1 : 10. An aliquot of 180 *μ*L of freshly prepared FRAP reagent was mixed with 20 *μ*L of extraction solution, and the reaction solution was protected from light for 30 min at 25°C prior to the determination of the absorbance at 593 nm. The extraction solvent was used instead ofthe sample solution as a blank.

#### 2.6.4. Cupric Ion Reducing Antioxidant Capacity (CUPRAC)

The CUPRAC of the extracts was carried out according to Reşat's method with modifications [31]. Firstly, reagents including CuCl_2_ (1 mL, 10 mM), neocuproine (1 mL, 7.5 mM), and ammonium acetate (pH 7.0) were fully mixed. Then, a sample (0.1 mL) was added to the blend, and 1 mL of UP water was employed to adjust the final volume to 4.1 mL. Next, the mixture was allowed to keep at room temperature for 30 min to complete the reaction. Finally, the absorbance of 0.2 mL of the reaction solution was measured at a wavelength of 450 nm. The sample solution was replaced with the extraction solvent as a blank.

#### 2.6.5. Total Antioxidant Capacity Assay

The total antioxidant capacity of the extracts was evaluated using the phosphomolybdenum method by Zengin and Aktumsek [32]. 0.1 mL of the sample solution was combined with 4 mL of the reagent solution containing 0.6 M sulfuric acid, 28 mM sodium phosphate, and 4 mM ammonium molybdate. The mixture was allowed to incubate in a water bath for 90 min at 95°C. After the reaction, the mixture was rapidly cooled with running water, and the sample absorbance was measured at 695 nm. 0.1 mL of the extraction solvent was used instead of the sample solution as a blank.

#### 2.6.6. Reducing Power

The test was performed based on a method from Sun et al. with slight modifications [33]. 1.0 mL of the sample extract, 2.5 mL of 0.2 M PBS (pH 6.6), and 2.5 mL of 1% potassium ferricyanide solution were mixed well, next, the mixture was kept in a water bath at 50°C for 20 min. After the reaction was completed, the mixed solution was quickly cooled by running water, then 2.5 mL of 10% trichloroacetic acid solution was added, and it was allowed to stand at room temperature for 10 min. After that, 5 mL of the above reaction solution was thoroughly mixed with 5 mL of ultrapure water and 1 mL of 0.1% ferric chloride solution, and the mixture was kept for another 10 min. Finally, the absorbance value of the reaction solution was measured at 700 nm. The absorbance measured by the extraction solvent instead of the sample solution was blank.

### 2.7. Enzyme Inhibition Assays

The enzyme inhibitory activity of the evaluated samples is expressed as IC_50_ values by GraphPad Prism v8.0.

#### 2.7.1. Tyrosinase

For the tyrosinase inhibition assay, a previously reported method was employed with slight modifications [34]. In short, 30 *μ*L of sample solution, 40 *μ*L of 1 mg·mL^−1^ L-DOPA solution, and 50 *μ*L of phosphate buffer (50 mM, pH 6.8) were added to a 96-well plate. Furthermore, the mixture was preincubated at 25°C for 15 min. Subsequently, 40 *μ*L of tyrosinase (800 U·mL^−1^) solution was added, and the reaction mixture was treated at 25°C for 15 min. After the incubation, a wavelength of 478 nm was used to determine the absorbance of the blend. The inhibition (%) of test samples on tyrosinase could be calculated as follows:

(3) $$\begin{array}{c} \text{Tyrosinase inhibition}\left(\%\right)=\left(1-\frac{A_{1}-A_{2}}{A_{3}-A_{4}}\right)\times 100\%, \end{array}$$

where *A*_1_ is the absorbance of the tested sample with enzyme, *A*_2_ is the absorbance of the sample blank without enzyme, *A*_3_ is the absorbance of the control sample without test sample, and *A*_4_ is the absorbance of the control blank without test sample and enzyme, respectively.

#### 2.7.2. *α*-Glucosidase

The *α*-glucosidase inhibition activity of the samples was measured using PNPG as a substrate based on the modified method of Li et al. [35]. Briefly, 20 *μ*L 100 mM phosphate buffer (pH 6.8), 20 *μ*L investigated sample, and 20 *μ*L 3.5 mM 4-nitrophenyl-*β*-D-glucopyranoside (PNPG) in phosphate buffer were sequentially added to each sample. After a 5 min preincubation at 37°C, 20 *μ*L 5 U·mL^−1^*α*-glucosidase in phosphate buffer was added to the mixture, which was mixed well to start the reaction. After incubation for 15 min at 37°C, the reaction was stopped by adding 80 *μ*L of 0.2 M sodium carbonate solution. Thereafter, the absorbance value at 405 nm was recorded. The *α*-glucosidase inhibition activity of the samples was expressed as the percentage inhibition according to the following equation:

(4) $$\begin{array}{c} \alpha -\text{glucosidase inhibition}\left(\%\right)=\left(1-\frac{A_{1}-A_{2}}{A_{3}-A_{4}}\right)\times 100\%, \end{array}$$

where *A*_1_, *A*_2_, *A*_3_, and *A*_4_ are the absorbance of the tested sample, sample blank, control without the test sample, and control blank without the test sample and enzyme, respectively.

### 2.8. Statistical Analysis

Unless otherwise stated, all experiments were carried out in triplicate and data were reported as mean ± SD. In order to identify which method might be a suitable technique with good biological activity, one-way ANOVA followed by a Tukey's post hoc comparison test was conducted to characterize the extracts using SPSS 26.0 software. Differences were considered statistically significant when *p*-value <0.05. In order to establish the relationship between the tested phytochemical content and the evaluated biological activities, the Pearson correlation coefficients were carried out. Furthermore, multivariate analysis, principal component analysis (PCA), and hierarchical cluster analysis (HCA) were carried out to cluster the extracts obtained from different extraction methods by Origin (Version 2019b) in terms of biological activities.

## 3. Results

### 3.1. Identification of Flavonoids in the Analyzed *O. indicum* Extracts through UHPLC-DAD Orbitrap Elite MS

**Figure 1:**
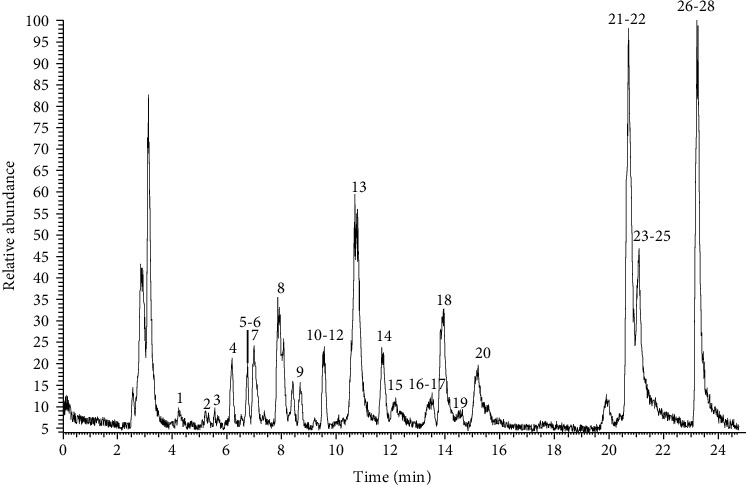
Representative TIC chromatography of the analyzed *O. indicum* extracts.

**Figure 2:**
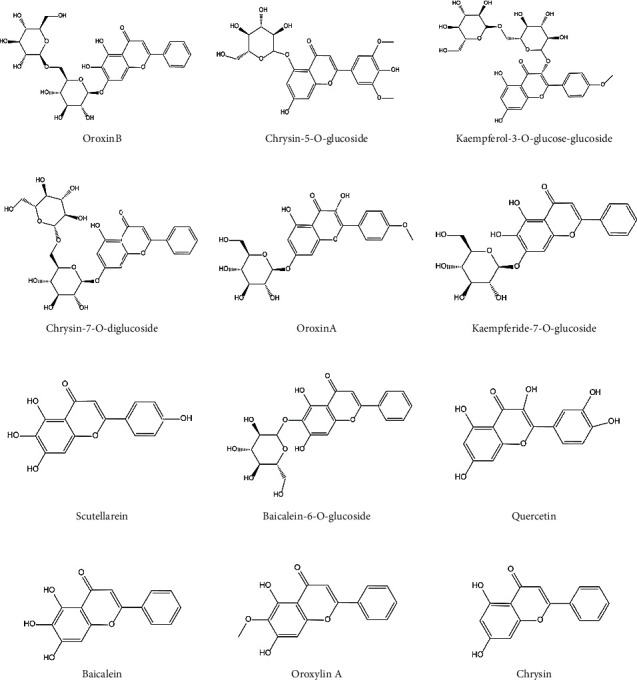
Chemical structures of major compounds.

**Figure 3:**
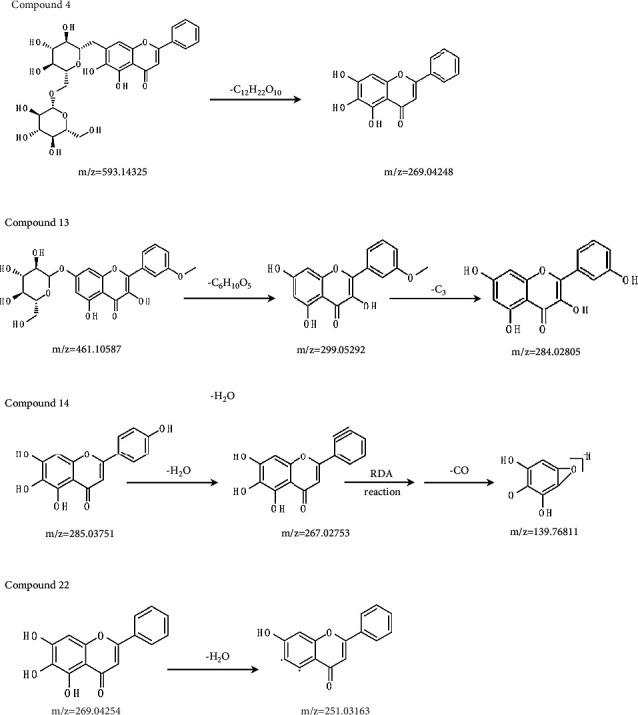
The predominant fragmentation pathway of representative flavonoids.

Identification of flavonoids was conducted by UHPLC-DAD Orbitrap Elite MS. In order to achieve a good separation of the studied compounds, the gradient elution program was optimized, and satisfactory results were obtained through the optimized gradient. A total of 28 flavonoids (Figure 1) are probationary identified from different extract samples based on the MS data, as well as comparison with literature data and reference standards. Among them, oroxin B, chrysin-5-O-glucoside, kaempferol-3-O-glucose-glucoside, chrysin-7-O-diglucoside, oroxin A, kaempferide-7-O-glucoside, scutellarein, baicalein-6-O-glucoside, quercetin, baicalein, oroxylin A, and chrysin were major compounds (Figure 2). The detailed information is shown in Table 1. The predominant fragmentation pathway of representative flavonoids is displayed in Figure 3.

The experiments were carried out in negative ion mode and all flavonoids analyzed showed a good fragmentation pattern and produced deprotonated molecules [M-H]^−^. Full MS/dd-MS2 mode collected all sample data, enabling the identification of targeted and untargeted compounds based on retention time, molecular ion (*m*/*z*), and MS^2^ fragments.

### 3.2. Quantification of Flavonoids in the Analyzed *O. indicum* Extracts

**Figure 4:**
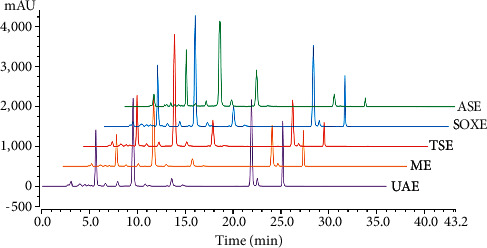
HPLC-DAD chromatograms referred to the 277 nm of different *O. indicum* extracts. The blue is Soxhlet extraction (SOXE), the purple is referred to ultrasound-assisted extraction (UAE), the orange to maceration extraction (ME), the red to tissue-smashing extraction (TSE), and the green is accelerated-solvent extraction (ASE).

A quantitative analysis of four main flavonoids based on the standard compounds was performed by UHPLC.  Figure 4 shows the UHPLC-DAD chromatogram referred to the 277 nm of different *O. indicum* extracts and the results are summarized in Table 2. As can be observed, SOXE extract displayed the highest oroxin B content, followed by ASE, UAE, TSE, and ME. In addition, ASE extract was found to possess the highest oroxin A content, recording 30926.33 ± 539.32 *μ*g·g^−1^. Regarding baicalein and chrysin contents, the UAE extract showed higher contents when compared to other extracts, being 6749.01 ± 118.31 and 3440.71 ± 15.40 *μ*g·g^−1^, respectively. Contrary to oroxin B and oroxin A contents, the contents of baicalein and chrysin extracted by ASE were the lowest. It was worth noting that SOXE also extracted relatively higher levels of baicalein and chrysin.

### 3.3. Bioactive Compounds in the Analyzed *O. indicum* Extracts

The information about the discrepancy in phytochemical composition of extracts obtained by different extraction techniques has been investigated infrequently. Therefore, *O. indicum* extracts obtained from different techniques were compared in terms of phytochemical content in this study. As shown in Table 3, the TPC of the extracts ranged from 15.05 ± 0.11 to 50.99 ± 1.78 mg GAE/g extract in the order of SOXE > ASE > UAE > TSE > ME. In addition, *O. indicum* extracts obtained by ASE (34.92 ± 0.38 mg RE/g extract) were characterized by the highest TFC, while the lowest TFC was recorded from extracts obtained by ME (20.74 ± 0.72 mg RE/g extract).

### 3.4. Antioxidant Activity of the Analyzed *O. indicum* Extracts

Given the complexity of phytochemicals, antioxidant capacities were evaluated by a variety of measurement methods targeting different mechanisms of action. In the current work, radical scavenging (ABTS and DPPH), reducing power (FRAP, CUPRAC, and potassium ferricyanide), and total antioxidant capacity assays were applied to provide a deep insight into the antioxidant capacity of *O. indicum* extracts obtained from different extraction methods. Data (mean ± SD) relating to the antioxidant ability of *O. indicum* extracts were expressed as mg of Trolox equivalent per g of extract. As may be seen in Table 4, assessment of both ABTS and DPPH radical scavenging activity of *O. indicum* extracts reveals that extracts produced by SOXE (27.58 ± 0.19 and 41.18 ± 0.77 mg TE/g, for ABTS and DPPH assays, respectively) were the most active, while extracts obtained by ME were the least active radical scavenger. FRAP, CUPRAC, and potassium ferricyanide assays employed to estimate the reducing power of studied extracts showed that SOXE extract (FRAP: 80.60 ± 0.68 mg TE/g; CUPRAC: 160.57 ± 0.83 mg TE/g; potassium ferricyanide: 129.99 ± 1.61 mg TE/g) possessed the highest reducing power compared to other extracts from different extraction techniques. Results of the total antioxidant potential (by phosphomolybdenum assay) are summarized in Table 4. It is worth highlighting that SOXE and ASE extracts showed significantly (*p* < 0.05) higher total antioxidant potential. Among *O. indicum* extracts, the lowest activity was observed in ME extract (52.79 ± 3.17 mg TE/g extract).

### 3.5. Enzyme Inhibitory Properties of the Analyzed *O. indicum* Extracts

Regarding the enzyme inhibitory potential of the extracts, two enzymes involved in type II diabetes and skin diseases, *α*-glucosidase and tyrosinase, were selected to evaluate the differences between different extracts. Results are presented in Table 5 with an IC_50_ value (mg·mL^−1^). Notably, all extracts showed inhibitory activities against the studied enzymes.

In terms of tyrosinase, the UAE extract showed the highest inhibition activity (IC_50_: 16.57 ± 0.53 mg·mL^−1^) followed by SOXE and TSE extracts (IC_50_: 19.80 ± 0.13 mg·mL^−1^ and 22.38 ± 1.07 mg·mL^−1^, respectively), then extract produced by ASE presented 29.34 ± 1.06 mg·mL^−1^ of IC_50,_ and the lowest inhibition activity was obtained using the ME technique (IC_50_: 33.45 ± 2.19 mg·mL^−1^). As for *α*-glucosidase, SOXE, UAE, and ME extracts exhibited a similar inhibitory activity with no significant differences in IC_50_ values. Different from obtained results of tyrosinase inhibitory activity, TSE extract expressed the lowest activity against *α*-glucosidase with IC_50_ value of 9.15 ± 0.09 mg·mL^−1^. Compared with the extracts obtained by four other extraction techniques, ASE technique (IC_50_: 4.17 ± 0.07 mg·mL^−1^) showed moderate inhibitory activity on *α*-glucosidase.

### 3.6. Correlation Analysis

In order to elucidate the possible contribution of phytochemical content to the biological properties observed when considering the different extraction methods, Pearson's correlation coefficients were calculated in this study. The result is summarized in Table 6. TPC had a significant positive correlation with all the six antioxidant indexes inspected (*p* < 0.01). the correlation coefficients of ABTS, DPPH, potassium ferricyanide, phosphomolybdenum, FRAP, and CUPRAC were 0.972, 0.917, 0.983, 0.686, 0.965, and 0.976, respectively. Similarly, a notable positive correlation was shown between TFC and the ABTS radical scavenging activity, DPPH, phosphomolybdenum, FRAP, and CUPRAC (at least (*p* < 0.05) with a correlation coefficient of 0.575, 0.814, 0.976, 0.666, and 0.656, respectively). However, when it comes to potassium ferricyanide, we found no significant correlation between TFC and potassium ferricyanide. The correlation coefficient was 0.511. Regarding enzyme inhibitory activities, TPC was found to be significantly and negatively correlated with tyrosinase inhibitory activity (*p* < 0.05) with a correlation coefficient of 0.562, while no significant correlations were observed between TFC and enzyme inhibitory activities.

### 3.7. Multivariate Analysis

**Figure 5:**
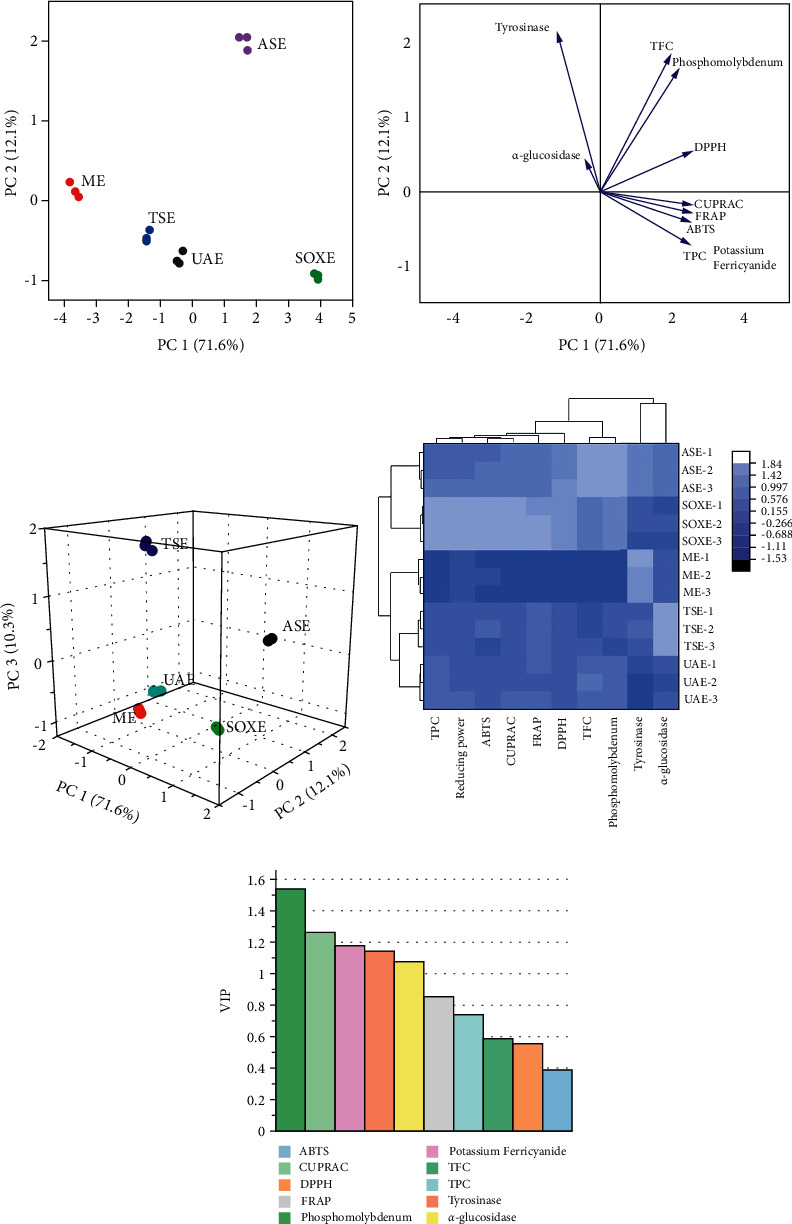
Multivariate analysis results. (a) The PCA score scatter plot of samples on the first two principal components showing cluster trends. (b) Loading plot of samples on the first two components displaying the relationship between the evaluated biological activities. (c) Three-dimensional score scatter plot of samples. (d) Heat Maps based on the studied biological activities. (e) VIP scores showing the best discriminating variables (bioactive compounds and biological activities) in the model (VIP scores which are higher than 1 was considered to be important).

Multivariate analysis, namely PCA and HCA, was carried out in order to provide a comprehensive understanding of the discrepancies between different extraction techniques applied in this study and to cluster these techniques according to evaluated biological substance content and biological activities. PCA score plot showed that five extraction techniques were effectively distinguished (Figures 5(a) and 5(c)). 94.0% of the sample variables could be explained by the first three components, among which SOXE and ASE were distinguished from UAE, TSE as well as ME on the first dimension. ASE and ME were distinguished from other three extraction methods on the second dimension, while TSE and ASE were distinguished from other extraction methods on the third dimension. Additionally, as shown in Figure 5(d), HCA displays three distinct groups, among which SOXE and ASE methods were grouped together, UAE and TSE methods were grouped together while only ME method was grouped separately. In fact, the differences between different extraction methods could be effectively characterized when it comes to the measured biochemical contents and biological activities evaluated in the study whilst some methods had similarity. Another information revealed by Figure 5(e) was that half of the biological activities determined were found to have the highest discriminating ability because the VIP score was higher than 1, namely phosphomolybdenum, CUPRAC, potassium ferricyanide, tyrosinase, and *α*-glucosidase. In particular, TFC, TPC, and antioxidant activities (Figures 5(a) and 5(b)) recorded for SOXE and ASE were the most potent among all the studied extraction methods, while ME showed better enzyme inhibitory activities. All the observed variations may arise from differences in extraction conditions. It revealed that different extraction techniques could impact on the biological activities of *O. indicum*. On the other hand, each technique had its own shortcomings and advantages.

## 4. Discussion

Providing comprehensive information on natural products is the foundation for the development of modern medicine and functional food [36]. In the present study, *O. indicum* was extracted using five different extraction methods: maceration extraction, Soxhlet extraction, ultrasound-assisted extraction, tissue-smashing extraction, and accelerated-solvent extraction. The phytochemical, antioxidant, and enzyme inhibitory activities of *O. indicum* extracts were assessed to determine the application prospect of *O. indicum* extract as a potential nutraceutical source.

Choosing an appropriate extraction method is the first critical step in the analysis of natural products. Traditionally, ME and SOXE are widely applied to extract plant components from natural products [37]. However, they are time-consuming, labor-intensive, require large amounts of extraction solvents and have low extraction yield [38, 39]. Therefore, in this case, nonconventional extraction techniques can be applied to extract phytochemical components from natural products [40]. UAE, TSE, and ASE have been widely applied to extract flavonoids as an alternative to conventional extraction techniques because of the advantages of short extraction time, less solvent consumption, and environmental friendliness, which have attracted attention [39, 40].

The phytochemical components in the extracts of *O. indicum* differed according to different extraction methods. *O. indicum* is rich in flavonoids, thus the flavonoids in the extracts of *O. indicum* obtained by five different extraction methods were identified through UHPLC-DAD Orbitrap Elite MS, and a total of 28 flavonoids were identified. *O. indicum* is reported to contain important flavonoids, namely oroxin A, oroxin B, baicalein, and chrysin [13]. These compounds have been extensively reported to possess various biological activities. Oroxin A, oroxin B, and chrysin are affirmed for their antioxidant, anticancer, and anti-inflammatory activities [41–43]. As for baicalein, many biochemical activities have been assessed, like anticancer, anti-inflammatory, antibacterial, antihyperglycemia, neurogenesis, cardioprotective, antiadipogenesis, and antioxidant activities [44]. Given the remarkable activities of these compounds, quantitative analysis was carried out. The results showed that oroxin A and oroxin B have the highest content among the extracts of *O. indicum* (range 8372.31 to 31465.65 *μ*g·g^−1^). The methods using high temperatures, namely, SOXE and ASE, seemed to extract higher contents concerning the content of oroxin A and oroxin B, while UAE using low temperatures extracted higher levels of baicalein and chrysin. Furthermore, the UAE and TSE extracts showed a slightly lower level of oroxin A and oroxin B than those of SOXE and ASE extracts. It was worth noting that SOXE also extracted relatively higher baicalein and chrysin, which might be attributed to the longer extraction time. The content of baicalein and chrysin obtained by ASE was the lowest, which might be due to the destruction of these two components under the high temperature and pressure extraction conditions of ASE. In summary, SOXE and ASE are suitable for the extractions of oroxin A and oroxin B, while UAE and SOXE might be the best options for collecting baicalein and chrysin.

Phenolic and flavonoids compounds are secondary metabolites that are widely found in plants, which have been claimed to possess a variety of biological activities that play an important role in health-promoting and nutraceutical potential of plants and food [45]. Thus, we conducted an analysis with regard to TPC and TFC in *O. indicum* extracts. The extraction of phenolic compounds depends on the temperature and the polarity of the solvent. Ethanol was used as the extraction solvent in the present research for the reason that many studies have confirmed that alcoholic solvents are used to extract phenolic components from natural products [46]. The findings concluded that all *O. indicum* extracts can be a rich source of phenols and flavonoids. Among them, TPC extracted by SOXE using high temperature was the highest while TFC extracted by ASE was the highest, so that these two extraction methods have been identified as an avenue for the better extraction of phenolic and flavonoids from plants. Flavonoids mostly belong to polyphenol compounds, thus TPC should be greater than TFC theoretically. However, different reference substances were used in the determination (gallic acid for TPC and rutin for TFC), and different extraction methods may lead to incomplete or destroyed extraction of polyphenols, thus TFC of ME and ASE extracts were slightly greater than TPC [10]. The finding corroborates with research performed by Zheleva-Dimitrova et al. [18], they concluded that SOXE and ASE were useful to extract phenolic and flavonoids compounds from plants. However, one of the merits of ASE technique is that the time required for extraction was greatly reduced and the work efficiency was effectively improved.

Actually, total bioactive component content can affect the biological activities of plant extracts [24]. In the present study, radical scavenging (ABTS and DPPH), reducing power (FRAP, CUPRAC, and potassium ferricyanide), and phosphomolybdenum assays were applied to evaluate the antioxidant capacity of *O. indicum*. The same trend was observed for all antioxidant ability determinations conducted. Data amassed in the present study demonstrated that SOXE extract exhibited the highest antioxidant activity. Compared with SOXE, ASE extract showed higher total antioxidant potential, while other antioxidant activities were second only to SOXE. Regardless of the mechanism of antioxidant capacity, SOXE and ASE have the strongest activity, followed by UAE and TSE, and ME has the lowest antioxidant activity. It could be observed from the correlation analysis of Section 3.5 that TPC/TFC had a significant positive correlation with the antioxidant activity index, which implied that the antioxidant properties of *O. indicum* extracts were influenced by TPC/TFC. Since the extracts of SOXE and ASE showed the highest total bioactive component content, their antioxidant activities were notably higher than those of the other three extracts. Our findings are consistent with previous studies that revealed a high correlation between phenolic content and antioxidant capacity [47–49]. As a nonconventional extraction technology, ASE greatly shortened the extraction time compared with SOXE. Therefore, ASE was considered to be an effective method for extracting bioactive compounds.

Diabetes is a major public health problem that affects the quality of human life and threatens human life, of which type 2 diabetes accounts for about 90% [50]. Inhibition of a key enzyme (*α*-glucosidase) is usually used as a treatment strategy for diabetes. However, acarbose, which is currently available for the treatment of diabetes, can cause adverse reactions in patients, including gastrointestinal reactions, hypoglycemia, and hepatotoxicity, which limits its application [51]. Similarly, existing tyrosinase inhibitors (such as arbutin and kojic acid), which regulate the synthesis of melanin to treat pigmentation, have been found to have poor stability, low activity, and cause adverse reactions [52]. Therefore, there is an urgent need to find new inhibitors with low adverse reactions from natural products, especially TCM. Thus, this study conducted inhibitory activity experiments on *α*-glucosidase and tyrosinase. The extraction methods seemed to affect the enzyme inhibitory activities. All the studied extracts showed inhibitory properties on *α*-glucosidase. UAE, ME, and SOXE extracts showed the highest inhibitory activity (IC_50_ values range from 1.23 to 1.35 mg·mL^−1^) with no significant difference in *α*-glucosidase inhibitory effects. Despite its lower TPC and TFC contents compared with other extraction methods, the ME extract had high activity against *α*-glucosidase. The antidiabetic pharmacological effect of *O. indicum* might be attributed to the content of oroxin A and baicalein [44]. Sun et al. [53] have concluded that oroxin A from *O. indicum* prevented the progression from prediabetes to diabetes in streptozotocin and high-fat diet-induced mice. Zhang et al. [54] have proved that the antidiabetic effect of baicalein was associated with the modulation of gut microbiota in streptozotocin and high-fat-diet-induced diabetic rats. In addition, all extracts exhibited a significant inhibitory effect on tyrosinase, in the following: UAE > SOXE > TSE > ASE > ME. Correlation analysis showed that TPC was only negatively correlated with tyrosinase, while TFC had no significant correlation with the inhibitory activity of either enzyme. It has been previously reported that the enzyme inhibitory activity was not related to TPC/TFC [55]. From this perspective, the observed enzyme inhibitory abilities may be related to the presence of nonphenolic compounds, which may contribute to enzyme inhibitory potential.

The observed discrepancy indicated that chemical components in the extracts may contribute to the biological effects in a synergistic manner owing to varying biological potentials between different phenolic classes. To sum up, our findings indicate that each extraction method is considered to be an effective extraction method, which can recover bioactive compounds of interest in the food, pharmaceutical, cosmetic, and nutraceutical fields from *O. indicum*.

## 5. Conclusion

Safe, efficient, and sustainable extraction techniques substituting conventional techniques are attracting increasing interest. However, the extraction technique should not affect the biological activities of the extract. In the present study, the phytochemical composition and bioactive properties (antioxidant and enzyme inhibitory activities) of *O. indicum* extracts obtained using conventional and nonconventional techniques were compared to illustrate the detailed information of the extracts and to provide a basis for further research and practical application. The finding suggested that the extracts enriched TPC/TFC obtained by SOXE and ASE showed robust antioxidant activity. In addition, the study supported that UAE and ME extracts possessed strong enzyme inhibitory activities in spite of the fact that the total amount of phytochemicals analyzed was slightly lower than those of SOXE and ASE extracts. Various factors including the solvent, time, and temperature used in the extraction may influence the extraction of phytochemicals in plant materials. Therefore, it is essential to choose the most suitable conditions for extraction depending on the purpose of application to increase the potential application of *O. indicum* in the pharmaceutical, food, or cosmetic industries.

## Figures and Tables

**Table 1:** UHPLC-DAD-MS qualitative analysis of flavonoids compounds in *O. indicum* extracts.

7F0E0No	*t *_*R*_ (min)	Compound name	Molecular formula	Mw	Molecular ion (*m*/*z*)	MS^2^ fragments (*m*/*z*)
1	4.09	Scutellarein-5-O-gentiobiose	C_27_H_30_O_16_	610	609.13947	285.03723, 267.37967
2	5.37	Scutellarein-7-O-glucoside	C_21_H_20_O_11_	448	447.08804	285.03732
3	5.55	Hyperoside	C_21_H_20_O_12_	464	463.08325	300.02444, 273.03748, 178.99692
4	6.25	Oroxin B^*∗*^	C_24_H_30_O_12_	594	593.14325	269.04248, 251.02994
5	6.84	Chrysin-5-O-glucoside	C_21_H_20_O_9_	416	415.09845	253.04776
6	6.86	Quercetin-3-rhamnoside	C_20_H_18_O_16_	434	433.07303	300.02438, 285.27863, 178.99696
7	7.04	Kaempferol-3-O-glucose-glucoside	C_28_H_32_O_16_	624	623.15857	299.05283, 284.02896
8	8.03	Chrysin-7-O-diglucoside	C_27_H_30_O_14_	578	577.14893	253.04778
9	8.96	Oroxylin A-7-O-glucose-glucoside	C_28_H_32_O_15_	608	607.12329	193.03336
10	9.47	Oroxin A^*∗*^	C_21_H_20_O_10_	432	431.09409	269.04266
11	9.65	Baicalin	C_21_H_18_O_11_	446	445.07242	269.04242
12	9.69	Baicalein-6-O-glucoside	C_21_H_20_O_10_	432	431.09323	269.04251, 223.94516
13	10.69	Kaempferide-7-O-glucoside	C_22_H_22_O_11_	462	461.10587	299.05292, 284.02805, 136.98642
14	11.68	Scutellarein	C_15_H_10_O_6_	286	285.03751	267.02753, 139.76811
15	12.77	Chrysin-7-O-glucoside	C_21_H_20_O_19_	416	415.09836	253.04799, 299.05283
16	13.33	Chrysin-7-O-*β*-D-glucuronid	C_21_H_18_O_10_	430	429.07794	253.04779, 175.02310, 113.02351
17	13.40	Baicalein-7-O-rhamnoside	C_20_H_18_O_9_	402	401.08322	269.04263
18	13.67	Baicalein-6-O-glucoside	C_21_H_20_O_10_	432	431.09348	269.04254, 284.39246
19	14.73	Wogonoside	C_22_H_20_O_11_	460	459.08981	283.05826, 268.03427
20	15.52	Quercetin^*∗*^	C_15_H_10_O_7_	302	301.03204	273.03763, 257.04340, 151.00235
21	20.44	Dihydrobaicalein	C_15_H_12_O_5_	272	271.05795	253.04767, 197.05905, 125.02325
22	20.85	Baicalein^*∗*^	C_15_H_10_O_5_	270	269.04254	251.03163, 225.05370, 197.05875, 169.06404
23	21.15	Oroxylin A^*∗*^	C_16_H_12_O_5_	284	284.02930	268.03503, 240.04051, 136.98694
24	21.32	Hispidulin	C_16_H_12_O_6_	300	299.05273	284.02957
25	22.01	Kaempferide	C_16_H_12_O_6_	300	299.05255	284.02936, 227.69331
26	23.40	Chrysin^*∗*^	C_15_H_10_O_4_	254	253.04953	209.06033, 143.05006
27	23.48	Wogonin	C_16_H_12_O_5_	284	283.05823	268.03500
28	23.50	Apigenin	C_15_H_10_O_5_	270	268.03430	117.03394

^*∗*^Compound identified by comparison with the standard substance.

**Table 2:** Quantitative analysis results of four main flavonoids in *O. indicum* extracts.

Analytes	Regression equation	*R * ^2^	Linear range (*μ*g·mL^−1^)	LODs (*μ*g·mL^−1^)	LOQs (*μ*g·mL^−1^)	Extraction methods	Mass fraction (*μ*g·g^−1^)	Precision (*n* = 6, RSD%)	Stability (*n* = 6, RSD%)	Repeatability (*n* = 6, RSD%)
Oroxin B	*Y* = 0.2077*X* − 0.1083	0.9999	2–250	0.03	0.1	UAE	15348.66 ± 181.81^c^	0.22	0.84	0.34
						ME	8470.05 ± 97.74^e^	0.30	1.00	0.29
						TSE	13120.20 ± 79.83^d^	0.25	0.89	0.65
						SOXE	16243.03 ± 45.72^a^	0.30	0.54	0.42
						ASE	15765.67 ± 222.87^b^	0.62	0.28	1.23

Oroxin A	*Y* = 0.303*X* − 0.1537	1	2–250	0.03	0.1	UAE	15545.80 ± 77.63^d^	0.10	0.35	0.30
						ME	11849.79 ± 62.58^e^	0.43	0.44	0.36
						TSE	20000.39 ± 163.69^c^	0.25	0.94	0.38
						SOXE	20265.19 ± 131.16^b^	0.14	0.35	0.54
						ASE	30926.33 ± 539.32^a^	0.25	0.26	1.38

Baicalein	*Y* = 0.5496*X* − 0.1321	0.9999	1–125	0.12	0.33	UAE	6749.01 ± 118.31^a^	0.09	1.20	0.28
						ME	3098.28 ± 10.93^d^	0.25	0.49	0.19
						TSE	3312.59 ± 69.43^c^	0.24	2.06	0.61
						SOXE	6129.22 ± 60.54^b^	0.13	0.57	0.60
						ASE	1083.34 ± 18.47^e^	0.48	0.86	1.78

Chrysin	*Y* = 0.6757*X* + 0.1699	0.9999	1–125	0.01	0.03	UAE	3440.71 ± 15.40^a^	0.31	0.31	0.38
						ME	1825.45 ± 8.05^c^	0.29	0.33	0.25
						TSE	1184.53 ± 5.16^d^	0.20	0.39	1.55
						SOXE	2618.77 ± 14.89^b^	0.14	0.56	0.42
						ASE	474.30 ± 8.61^e^	0.26	0.37	1.50

*Y* = peak area and *X* = concentration (*μ*g·mL^−1^). LOD: limit of detection (S/N = 3); LOQ: limit of quantification (S/N = 10). Mass fractions expressed are means ± S.D. of three parallel measurements. UAE: ultrasound-assisted extraction; ME: maceration extraction; TSE: tissue-smashing extraction; SOXE: Soxhlet extraction; and ASE: accelerated-solvent extraction. Statistical evaluation was carried out by one-way ANONA test. Different letters indicate significant differences between the tested extracts (*p* < 0.05).

**Table 3:** Total bioactive components of *O. indicum* extracts.

Extraction methods	Total phenolic contents (mg GAE/g extract)	Total flavonoid contents (mg RE/g extract)
UAE	30.11 ± 0.36^c^	27.44 ± 0.67^b^
ME	15.05 ± 0.11^e^	20.74 ± 0.72^d^
TSE	25.14 ± 0.11^d^	23.02 ± 0.61^c^
SOXE	50.99 ± 1.78^a^	28.58 ± 0.29^b^
ASE	32.81 ± 0.32^b^	34.92 ± 0.38^a^

Values expressed are means ± S.D. of three parallel measurements. RE: rutin equivalent; GAE: gallic acid equivalent. UAE: ultrasound-assisted extraction; ME: maceration extraction; TSE: tissue-smashing extraction; SOXE: Soxhlet extraction; and ASE: accelerated-solvent extraction. Statistical evaluation was carried out by one-way ANONA test. Different letters indicate significant differences between the tested extracts (*p* < 0.05).

**Table 4:** Antioxidant properties and total antioxidant capacity of *O. indicum* extracts.

Extraction methods	ABTS (mg TE/g extract)	DPPH (mg TE/g extract)	CUPRAC (mg TE/g extract)	FRAP (mg TE/g extract)	Phosphomolybdenum (mg TE/g extract)	Potassium ferricyanide (mg TE/g extract)
UAE	20.30 ± 0.57^c^	28.11 ± 0.49^c^	85.02 ± 3.63^c^	55.31 ± 0.91^c^	103.63 ± 5.17^c^	67.48 ± 2.9^c^
ME	17.63 ± 0.35^d^	20.78 ± 0.81^d^	52.04 ± 1.44^d^	35.80 ± 0.32^d^	52.79 ± 3.17^e^	45.94 ± 0.36^d^
TSE	20.19 ± 1.01^c^	26.68 ± 0.69^c^	79.01 ± 3.79^c^	53.44 ± 2.57^c^	82.72 ± 2.97^d^	64.62 ± 0.36^c^
SOXE	27.58 ± 0.19^a^	41.18 ± 0.77^a^	160.57 ± 0.83^a^	80.60 ± 0.68^a^	137.79 ± 0.35^b^	129.99 ± 1.61^a^
ASE	22.74 ± 0.63^b^	37.91 ± 1.15^b^	116.25 ± 0.80^b^	65.34 ± 1.42^b^	177.77 ± 1.02^a^	81.39 ± 1.22^b^

Values expressed are means ± S.D. of three parallel measurements. TE: trolox equivalent. UAE: ultrasound-assisted extraction; ME: maceration extraction; TSE: tissue-smashing extraction; SOXE: Soxhlet extraction; ASE: accelerated-solvent extraction. Statistical evaluation was carried out by one-way ANONA test. Different letters indicate significant differences between the tested extracts (*p* < 0.05).

**Table 5:** Enzyme inhibitory properties of the tested extracts from *O. indicum*.

Extraction methods	Tyrosinase (IC_50_ mg/mL)	*α*-glucosidase (IC_50_ mg/mL)
UAE	16.57 ± 0.53^a^	1.25 ± 0.07^a^
ME	33.45 ± 2.19^d^	1.35 ± 0.05^a^
TSE	22.38 ± 1.07^b^	9.15 ± 0.09^c^
SOXE	19.80 ± 0.13^b^	1.23 ± 0.09^a^
ASE	29.34 ± 1.06^c^	4.17 ± 0.07^b^

Values expressed are means ± S.D. of three parallel measurements. UAE: ultrasound-assisted extraction; ME: maceration extraction; TSE: tissue-smashing extraction; SOXE: Soxhlet extraction; and ASE: accelerated-solvent extraction. Statistical evaluation was carried out by one-way ANONA test. Different letters indicate significant differences between the tested extracts (*p* < 0.05).

**Table 6:** Matrix for correlation analysis between TE and IC_50_ value of evaluated biological activities and tested phytochemical content.

Biological activities	TPC	TFC	
				mg GAE/g extract	Correlation	mg RE/g extract	Correlation
Antioxidant properties(mg TE/g extract)	ABTS	UAE	20.87	UAE (30.47)	0.972^*∗∗*^	UAE (28.11)	0.575^*∗*^
		ME	17.98				
		TSE	21.20				
		SOXE	27.77				
		ASE	28.37				
	DPPH	UAE	28.60		0.917^*∗∗*^		0.814^*∗∗*^
		ME	21.59				
		TSE	27.37				
		SOXE	41.95	ME (15.16)		ME (21.46)	
		ASE	39.06				
	Potassium ferricyanide	UAE	70.38		0.983^*∗∗*^		0.511
		ME	46.30				
		TSE	64.98				
		SOXE	131.60				
		ASE	82.61				
	Phosphomolybdenum	UAE	108.80		0.686^*∗∗*^		0.976^*∗∗*^
		ME	55.96	TSE (25.25)		TSE (23.63)	
		TSE	85.69				
		SOXE	138.14				
		ASE	178.79				
	FRAP	UAE	56.22		0.965^*∗∗*^		0.666^*∗∗*^
		ME	36.12				
		TSE	56.01				
		SOXE	81.28				
		ASE	66.76	SOXE (52.77)		SOXE (28.87)	
	CUPRAC	UAE	88.65		0.976^*∗∗*^		0.656^*∗∗*^
		ME	53.48				
		TSE	82.80				
		SOXE	161.40				
		ASE	117.05				
Enzyme inhibitoryproperties (IC_50_ mg/mL)	Tyrosinase	UAE	16.04		−0.562^*∗*^		−0.141
		ME	31.26				
		TSE	21.31	ASE (31.13)		ASE (35.30)	
		SOXE	19.67				
		ASE	28.28				
	*α*-glucosidase	UAE	1.18		−0.266		−0.111
		ME	1.30				
		TSE	9.06				
		SOXE	1.14				
		ASE	4.10				

^*∗∗*^Correlation is significant at the 0.05 level (2-tailed). ^*∗∗*^Correlation is significant at the 0.01 level. TE: Trolox equivalent. IC_50_: the concentration of extracts (mg/mL) when the enzyme inhibitory activity reaches 50%.

## Data Availability

The datasets used and analyzed during this study are available from the corresponding author upon reasonable request.
